# Upregulation of ERp57 promotes clear cell renal cell carcinoma progression by initiating a STAT3/ILF3 feedback loop

**DOI:** 10.1186/s13046-019-1453-z

**Published:** 2019-10-30

**Authors:** Yan Liu, Jian-Xing Wang, Zi-Yuan Nie, Yue Wen, Xin-Ju Jia, Li-Na Zhang, Hui-Jun Duan, Yong-Hong Shi

**Affiliations:** 1 Department of Pathology, Hebei Medical University, 361 Zhongshan East Road, Shijiazhuang, 050017 People’s Republic of China; 2 Department of Anesthesiology, The 4th Hospital of Hebei Medical University, 169 Tianshan Street , 050000, Shijiazhuang, People’s Republic of China; 3 Department of Otolaryngology, The Second Hospital of Hebei Medical University, 215 Heping West Road Shijiazhuang, 050000 Shijiazhuang, People’s Republic of China; 4 Department of Hematology, The Second Hospital of Hebei Medical University, 215 Heping West Road Shijiazhuang, 050000 Shijiazhuang, People’s Republic of China; 5 Department of Endocrinology, The First Hospital of Hebei Medical University, 89 Donggang Road Shijiazhuang, 050000 Shijiazhuang, People’s Republic of China

**Keywords:** Clear cell renal carcinoma, ERp57, ILF3, STAT3, Proliferation

## Abstract

**Background:**

ERp57 dysfunction has been shown to contribute to tumorigenesis in multiple malignances. However, the role of ERp57 in clear cell renal carcinoma (ccRCC) remains unclear.

**Methods:**

Cell proliferation ability was measured by MTT and colony forming assays. Western blotting and quantitative real-time PCR (qRT-PCR) were performed to measure protein and mRNA expression. Co-immunoprecipitation (CoIP) and proximity ligation assay (PLA) were performed to detect protein-protein interaction. Chromatin immunoprecipitation (ChIP), ribonucleoprotein immunoprecipitation (RIP), and oligo pull-down were used to confirm DNA–protein and RNA–protein interactions. Promoter luciferase analysis was used to detect transcription factor activity.

**Results:**

Here we found ERp57 was overexpressed in ccRCC tissues, and the higher levels of ERp57 were correlated with poor survival in patients with ccRCC. In vivo and in vitro experiments showed that ccRCC cell proliferation was enhanced by ERp57 overexpression and inhibited by ERp57 deletion. Importantly, we found ERp57 positively regulated ILF3 expression in ccRCC cells. Mechanically, ERp57 was shown to bind to STAT3 protein and enhance the STAT3-mediated transcriptional activity of ILF3. Furthermore, ILF3 levels were increased in ccRCC tissues and associated with poor prognosis. Interestingly, we revealed that ILF3 could bind to ERp57 and positively regulate its expression by enhancing its mRNA stability. Furthermore, ccRCC cell proliferation was moderated via the ERp57/STAT3/ILF3 feedback loop.

**Conclusions:**

In summary, our results indicate that the ERp57/STAT3/ILF3 feedback loop plays a key role in the oncogenesis of ccRCC and provides a potential therapeutic target for ccRCC treatment.

## Background

Kidney cancer is 6th most frequently occurring cancer in men and 9th in women, with 65,340 new cases and 16,970 deaths reported in the United States in 2018 [1]. Clear cell renal cell carcinoma (ccRCC) represents the major subtype of renal cell carcinoma, accounting for 70% of kidney cancers [2]. Early-stage ccRCC patients benefit from the surgery. However, some ccRCC patients are asymptomatic until advanced stages with distance metastases, which have a 5-years survival of less than 20% [3, 4]. As a gene-driven malignancy, many genes have been identified that are involved in the development of ccRCC [5]. However, the basic molecular pathways involved in the development ccRCC remain unclear. Therefore, understanding the molecular mechanisms and identifying therapeutic targets of ccRCC is desirable.

ERp57, also known as GRp58 or PDIA3, is a member of the protein disulfide isomerase (PDI) gene family and is first reported in response to glycoprotein folding in endoplasmic reticulum (ER) [6, 7]. ERp57 contains extensive functions beyond its abilities in the ER. For example, in the cell membrane, ERp57 acts as a membrane receptor for 1α, 25-dihydroxy-vitamin D3 [8]. In the cytoplasm, ERp57 co-localizes with NF-κB, ATRA–RARα, and mTOR, forming a complex that has been implicated in various developmental processes [9, 10]. In the nucleus, ERp57 directly interacts with DNA or enhances the DNA-binding of the signal transducer and activator of transcription 3 (STAT3) complex, influencing binding of the transcription factor to DNA, and facilitating nuclear import and export of transcription factor [11]. Therefore, the various subcellular localizations and binding partners of ERp57 affect numerous physiological processes and diseases. A previous study reported that ERp57 is dysregulated in many types of cancer, and upregulation or downregulation of ERp57 is correlated with poor prognosis [12–14]. Interestingly, ERp57 is downregulated in early-stage cervical cancer, whereas it is highly expressed in invasion-stage cervical cancer, suggesting that the role of the ERp57 in cancer is complex and closely related to its molecular partner [9, 15]. However, the expression and function of ERp57 in ccRCC remains unclear.

Interleukin enhancer-binding factor 3 (ILF3) is formed by alternative splicing of the *Ilf3* gene and contains double-stranded RNA (dsRNA)-binding motifs (dsRBMs) and a RGG domain that is responsible for its association with AU-rich elements [16]. Previous studies have found that ILF3 was dysregulated in breast tumor, hepatocellular carcinoma, non-small cell lung carcinoma and ovarian cancer [17–20], indicating its potential functions in oncogenesis. For example, ILF3 promotes hepatocellular carcinoma cell proliferation by binding to and stabilizing Cyclin E1 mRNA [18]. ILF3 also moderates RARP1 expression in hepatocellular carcinoma by stabilizing PARP1 mRNA by binding to its 3′ untranslated region (UTR) [21]. Another study also confirmed that ILF3 could bind to VEGF 3′UTR AREs and enhance mRNA stability in breast cancer [19]. ILF3 was also shown to blocks the microRNA binding site in the urokinase-type plasminogen activator (uPA) 3′UTR and promote breast cancer cell proliferation [22]. However, whether ILF3 regulates ccRCC proliferation and the underlying molecular mechanism involved remain unclear.

In the present study, we observed increased levels of ERp57 in ccRCC tissue, and higher levels of ERp57 or ILF3 were correlated with poor patient survival. Moreover, overexpression of ERp57 induced ccRCC proliferation in vitro and in vivo. Importantly, we demonstrated protein interaction between ERp57 and STAT3, forming a complex that transcriptionally regulates ILF3 expression. In addition, ILF3 may bind to ERp57 3’UTR and positively regulate ERp57 expression by enhancing its mRNA stability. Taken together, our results indicate that the ERp57/STAT3/ILF3 feedback loop plays a key role in the proliferation mechanism of ccRCC and provides a potential therapeutic target for ccRCC treatment.

## Methods

### Tumor tissues and cell lines

ccRCC tissues and pathologically non-tumorous tissue were collected from the ccRCC patients at the Fourth Hospital of Hebei Medical University from July 2016 to June 2017. The protocol of this study was approved by the Ethics Committee of Hebei Medical University and written consent was obtained from each patient. All samples were immediately frozen in liquid nitrogen after surgery and then later stored at − 80 °C for further use.

Human ccRCC cell lines (SW839, A498, Caki1, 786–0, OSRC-2 and ACHN) were obtained in our lab. All cell lines were cultured in Dulbecco’s Modified Eagle’s Medium-high glucose (Gibco, USA) containing 10% fetal bovine serum (FBS) at 37 °C in an atmosphere of 5% CO_2_.

### Cell transfection

Lipofectamine 2000 (Invitrogen) was used for cell transfection according to the manufacturer’s protocols. The ERp57-shRNAs, ILF3-shRNAs and shRNA negative controls were designed by GenePharma Co., Ltd. (Shanghai, China). The overexpression plasmids of ILF3, ERp57 and luciferase assay plasmids was purchased from GENEWIZ Company (Suzhou, China).

### Quantitative real-time PCR (qRT-PCR)

RNA Purification Kit (RNAeasy Mini Elute kit, QIAGEN) were used to prepare total RNAs from tissues and culture cells according to the manufacturer’s protocol. The concentration and purity of total RNA were measured by using Nanodrop spectrophotometer (Thermo Fisher). M-MLV First Strand Kit (Life Technologies) was used to reversed-transcript the RNA to cDNA. StepOne Plus real-time PCR system (Applied Bioscience) was used for qRT-PCR with the primer as following: ERp57-F:GCAATGATGGGCCTGTGAAG, ERp57-R:TCTTTGCTGAGCTTCTCGCC; β-catenin-F:ATGACTCGAGCTCAGAGGGT, β-catenin-R:ATTGCACGTGTGGCAAGTTC; Cyclin E1-F:ATACTTGCTGCTTCGGCCTT**;** Cyclin E1-R:TCAGTTTTGAGCTCCCCGTC**;** DKC1-F:CGGAAGTGGGGTTTAGGTCC; DKC1-R:TTGGCAGACTCATCCTGCTT**;** EGFR-F:AACCCCGAGGGCAAATACAG**;** EGFR-R:GGAGATCGCCACTGATGGAG**;** HIF1a-F:ACCTATGACCTGCTTGGTGC**;** HIF1a-R:GGCTGTGTCGACTGAGGAAA**;** ILF3-F:TCTCGAGCTCCTGTGTGAGA**;** ILF3-R:TCTTCCCGTTGCTGTCTGTC**;** P4HB-F:GCAAAATCAAGCCCCACCTG**;** P4HB-R:ACCATGGGGCATAGAACTCC**;** Nocth2-F:GGCCTCCTTCTCTTGCATGT**;** Nocth2-R:ATCTTCTGTGCAGTCAGCCC**;** PAX3-F:TGCCGTCAGTGAGTTCCATC**;** PAX3-R:GAAGGGACCTTGATCCGAGC**;** TGFB1-F:TGAGACTTTTCCGTTGCCG**;** TGFB1-R:ACCGGGGGTGTCTCAGTATC**;** VDR-F:GCGAAGCATGAAGCGGAAGGC**;** VDR-R:CATCTCCCGCTTCCTCTGCACTTC mRNAs were subjected to quantitative real-time polymerase chain reaction **(**qRT-PCR) using the Platinum SYBR Green qPCR Super Mix UDG Kit (Invitrogen) and the ABI 7500 FAST system (Life Technologies). All gene-expression levels were normalized to GAPDH and calculated using the 2^−ΔΔCt^ formula.

### Western blot

Cultured cells and tissues were lysed with RIPA buffer (Beibo, China) and then protein for further western blot following the protocol as describe previously. The antibodies were used in present study as following: anti-ERp57 (Abcam, ab13506), anti-ILF3 (Abcam, ab133354), anti-Cyclin E1 (Abcam, ab33911), anti-Cyclin D1 (Proteintech, 26,939–1-AP), anti-STAT3 (Abcam, ab68153), anti-pSTAT3 (Abcam, ab76315) and anti-β-actin (Abcam, ab6276).

### Cell proliferation assays

MTT assay and colony formation assay were used to detect cell viability. For MTT assay, cells were seeded on 96-well plates and then transfected with shERp57 or ERp57 overexpression vector plasmid for 24, 48 or 72 h. 20 μL of MTT reagent (5 mg/mL; Sigma-Aldrich, USA) was added into each well. After incubating for 3–4 h, we measured the absorbance at 495 nm by using a microplate reader (Thermo Fisher, USA). For colony formation assay, 100 cells/well culture cells were seeded into 6-well plates and culture for 1 week and then fixed with a glacial acetic acid/methanol solution. 0.5% crystal violet was used to stain the colonies. Colony numbers was counted under a microscope.

### Proximity ligation assay

The proximity ligation assay (PLA) was performed as described previously [23] . Briefly, A498 cells were seeded into 6 well chamber slides and cultured for 24 h. Then 4% paraformaldehyde were used to fixate the sliders. Anti-ERp57 and anti-STAT3 were used to stain the slides. Rabbit PLUS and Mouse MINUS Duolink in situ proximity ligation assay (PLA) kits were used to detect the interaction between the two proteins following the manufacturer’s protocols. Fluorescence was detected using a laser scanning confocal microscope.

### Co-immunoprecipitation assay

Co-immunoprecipitation analysis was performed as described previously. Briefly, cultured cells were lysed by RIPA and then lysates were immunoprecipitated with anti-ERp57 or anti-ILF3 for 1 h at 37 °C. Protein A-agarose were added to the lysates for incubating overnight. Next day, Protein A-agarose-antigen-antibody complexes were collected by centrifugation at 12,000 g for 2 min at 4 °C and immunoprecipitation-HAT buffer was used to washed complexes for 5 times. Western blot was used to detect the bound proteins.

### Biotin pull-down of RNA

Biotin pull-down analysis was used to detect the interaction between ILF3 protein and ERp57 mRNA as previously described [24]. In brief, cells were transfected with 4 μg biotin-labeled RNA for 24 h. And then cells were cross-linked with 1% formaldehyde in PBS and quenched with 0.125 M glycine. Then cells were resuspended in lysis buffer on ice for 10 min and were sonicated. The cell lysate was diluted in two times volume with hybridization buffer. Streptavidin Dynabeads (Life Technologies) were blocked for 2 h at 4 °C in lysis buffer containing 1 mg/ml yeast tRNA and 1 mg/mL BSA and wash twice with 1 mL lysis buffer. 100 μL washed/blocked Dynabeads was added, and the whole mix was then rotated for 30 min at 37 °C. Beads were captured by magnets (Life Technologies) and washed five times. Beads were then subjected to RNA elution with buffer.

### Animal experiment

All animal experiment protocol was approved by the Institutional Animal Care and Use Committee of Hebei Medical University (approval ID: HebMU 20,080,026). 4–6 weeks of BALB/c nude mice were purchased from Vital River Laboratory Animal Technology Co., Ltd. (Beijing, china). 5 × 10^6^ A498 cells stably knocked down ILF3 and ERp57 or knocked down both of them were mixed with 50% Matrigel matrix then this suspension was injected subcutaneously into the left dorsal flanks of nude mice. The length and width of mouse tumor were measured twice a week with calipers. At the end of the experiment, the mice were euthanized by Carbon dioxide asphyxiation. At last, the tumor tissues were fixed in 4% formalin solution or stored at − 80 °C for following experiment.

### Chromatin immunoprecipitation (ChIP) assay

The chromatin immunoprecipitation (ChIP) assay was performed as described previously [24]. In brief, A498 cells were treated with 1% formaldehyde to cross-link proteins with DNA. The cross-linked chromatin was then prepared and sonicated to an average size of 400–600 bp. The samples were diluted 10-fold and then precleared with protein A-agarose/salmon sperm DNA for 30 min at 4 °C. The DNA fragments were immunoprecipitated overnight at 4 °C with anti-STAT3, anti-ERp57 or anti-IgG (as negative control) antibodies. After cross-linking reversal, ERp57 and STAT3 on ILF3 promotor was examined. Results were determined by qRT-PCR with the following primers: ILF3-chip-F1:GGACAAAGCACTCGGTCACGGG; ILF3-chip-R1:GATGGAGAAACTGAGGCCCAGGG; ILF3-chip-F2:GCTCTTCTTGCTCCAAATCCTGG; ILF3-chip-R2:GCTTAACTAAACCCCACTGTCTTCCAGG; ILF3-chip-F3:CTGACCTCAAGTGATCCGCCCACC; ILF3-chip-R3:CTGGGCGACAGAGCGAGACTCTGTCT.

### RNA immunoprecipitation (RIP) assays

RIP was performed as described previously [23]. In brief, A498 cells was harvested and lysed in NETN buffer and then cells were used to conduct RIP experiments using the anti-ILF3 antibody or IgG, and the Dynabeads™ Protein G Immunoprecipitation Kit (10007D, Thermo Fisher) according to the manufacturer’s instructions. Then the beads were washed three times with NETN buffer and RNA was isolated by using RNA Purification Kit (RNAeasy Mini Elute kit, QIAGEN) according to the manufacturer’s protocol. The RNA fraction isolated by RIP was quantified by NanoDrop 2000 (Thermo-Fisher) and used for RT-qPCR with the following primers:

Cyclin E1-UTR-F:CGTGCGTTTGCTTTTACAGA; Cyclin E1-UTR-R:AGCACCTTCCATAGCAGCAT; ERp57-UTR-F:GGGCCGAGAGGACAGAATGG; ERp57-UTR-R:GCTGTTCTAATCACCAGGGTAGGCC; Cyclin D1-UTR-F:AGCGCTGTTTTTGTTGTGTG; Cyclin D1-UTR-R:TCATCCTGGCAATGTGAGAA; GAPDH-F:ATGAATGGGCAGCCGTTAGG; GAPDH-R:TGGAATTTGCCATGGGTGGA.

### Luciferase assay

Luciferase assay analysis was performed as described previously [24]. In brief, A498 cells were seeded into a 24-well plate, ILF3 reporter construct or the empty reporter vector was co-transfected with pWPI-STAT3 and pRL-TK, or co-transfected with pWPI-vector and pRL-TK. After 24 h of transfection, luciferase activity was measured using a Dual-Glo Luciferase Assay System (Promega, Madison, WI) with a Flash and Glow (LB955, Berthold Technologies) reader. The specific target activity was expressed as the relative activity ratio of firefly luciferase to Renilla luciferase.

### Immunofluorescence staining

Cells were fixed with 4% formaldehyde and pre-incubated with 10% normal goat serum (710,027, KPL, USA). Following incubating with primary antibodies anti-ILF3 (Abcam, ab89100) and anti-ERp57 (Abcam, ab13248) and fluorescent-labeled secondary antibodies, then DAPI (157,574, MB biomedical) was used to stain nuclear counter. Images were captured by confocal microscopy (DM6000 CFS, Leica) and processed by LAS AF software.

### Immunohistochemistry (IHC) analysis

Five-micrometer paraffin cross-sections of the tissues were deparaffinized in xylene solution and rehydrated by using gradient ethanol concentrations. Sections were subjected to antigen retrieval with citrate buffer. After hydrogen peroxide and protein blocking, the sections were incubated with primary antibody as following: anti-ERp57 (Abcam, ab13506) and anti-ILF3 (Abcam, ab13506) at 4 °C overnight, and then was incubated in streptavidin (HRP)-biotin labeled secondary antibody. Images were acquired using a Leica microscope (Leica DM6000B, Switzerland) and digitized with LAS V.4.4 (Leica).

### Statistical analysis

Data were presented as mean ± SEM. Student’s *t* test was used to analyze differences between two groups. Spearman’s correlation analysis was used to evaluate the correlation analysis. Values of *P <* 0.05 were considered statistically significant. Graphpad Prism 7.0 software was using to perform the statistical analysis (GraphPad Software, San Diego, CA, USA).

## Results

### ERp57 is upregulated in ccRCC tissues and contributes to poor prognosis

**Fig. 1:**
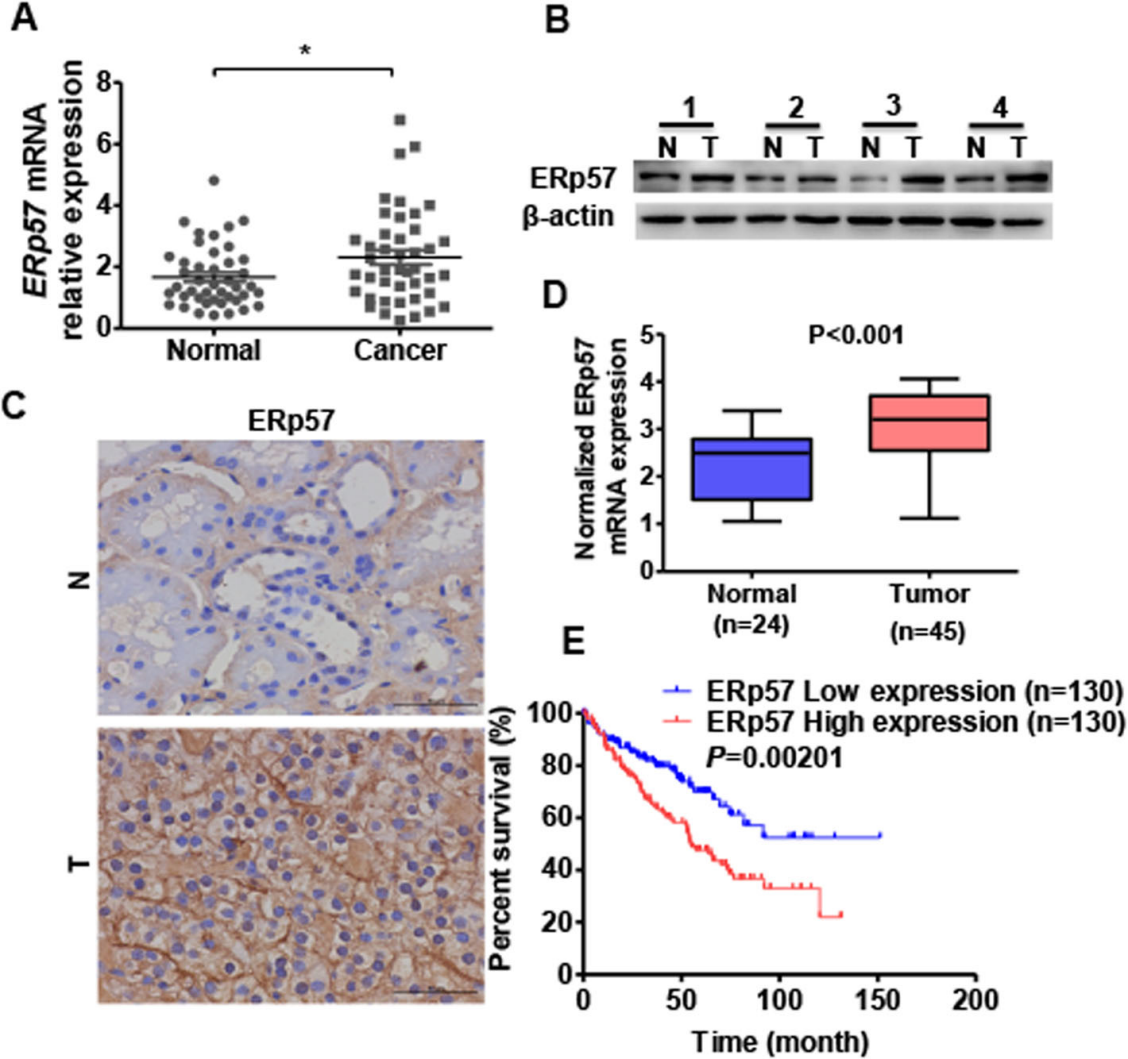
ERp57 is upregulated in ccRCC tissues and correlated with poor prognosis. **a** ERp57 mRNA level in ccRCC (*n* = 35) and normal kidney (*n* = 35) tissues was detected by qRT-PCR. Normalized against GAPDH. *P < 0.05 vs. normal kidney tissue. **b** ERp57 protein level in ccRCC tissues and normal kidney tissues were detected by western blot. **c** Immunohistochemistry stain was used to detected ERp57 protein level in ccRCC tissues and normal kidney tissues. Scale Bar = 50 μm. **d** ERp57 mRNA level in ccRCC tissues and normal kidney tissues analyzed in TCGA database. **d** Kaplan–Meier analysis was used to analyze the overall survival of ccRCC patients with low or high ERp57 level from TCGA database (cutoff value is 25%)

To identify the expression levels of ERp57 in ccRCC, we first measured its level in 35 samples of ccRCC tissues by qRT-PCR and Western blot analysis. The results showed that both mRNA and protein levels of ERp57 were frequently increased in ccRCC tissues (T) compared to normal kidney tissues (Fig. 1a and b). Similar results were observed in a cohort of ccRCC specimens by immunohistochemistry staining using an ERp57-specific antibody (Fig. 1c). In order to investigate the clinical significance of ERp57 in ccRCC, we analyzed the ERp57 mRNA expression level in 35 ccRCC and their clinicopathologic characteristics. The correlation analysis of ERp57 mRNA level significantly associated tumor size (Table 1). Moreover, we analyzed the ERp57 expression in ccRCC in a TCGA database and found that ERp57 mRNA levels were much higher in ccRCC tissues than in normal kidney tissues (Fig. 1d). Additionally, the TCGA database also revealed that higher ERp57 mRNA levels in patients with ccRCC were associated with poor overall survival (Fig. 1e). These results suggest that the upregulation of ERp57 promotes ccRCC progression.

**Table 1:** Clinicopathological Characteristics

Characteristics	ERp57 expression			ILF3 expression		
	Low (%)	High (%)	*P* value	Low (%)	High (%)	*P* value
Age						
≤ 56 years	9 (50.00)	9 (50.00)	0.738	8 (42.11)	11 (57.89)	0.505
> 56 years	7 (41.18)	10 (58.82)		9 (56.25)	7 (43.75)	
Gender						
Male	11 (45.83)	13 (54.17)	1.000	9 (39.13)	14 (60.87)	0.164
Female	5 (45.45)	6 (54.55)		8 (66.67)	4 (33.33)	
Tumor size						
≤ 7 cm	8 (80.00)	2 (20.00)	0.022	10 (76.92)	3 (23.08)	0.015
> 7 cm	8 (32.00)	17 (68.00)		7 (31.82)	15 (68.18)	
pT status						
pT_1_- pT_2_	7 (41.18)	10 (58.82)	0.738	9 (45.00)	11 (55.00)	0.738
pT_3_ -pT_4_	9 (50.00)	9 (50.00)		8 (53.33)	7 (46.67)	
pN status						
pN0	12 (52.17)	11 (47.83)	0.476	8 (38.10)	13 (61.90)	0.176
pN1 –pN3	4 (33.33)	8 (66.67)		9 (64.29)	5 (35.71)	
TNM stage						
I-II	9 (42.86)	12 (57.14)	0.739	10 (52.63)	9 (47.37)	0.738
III-IV	7 (50.00)	7 (50.00)		7 (43.75)	9 (56.25)	

### ERp57 plays a critical role in ccRCC cell proliferation and migration

**Fig. 2:**
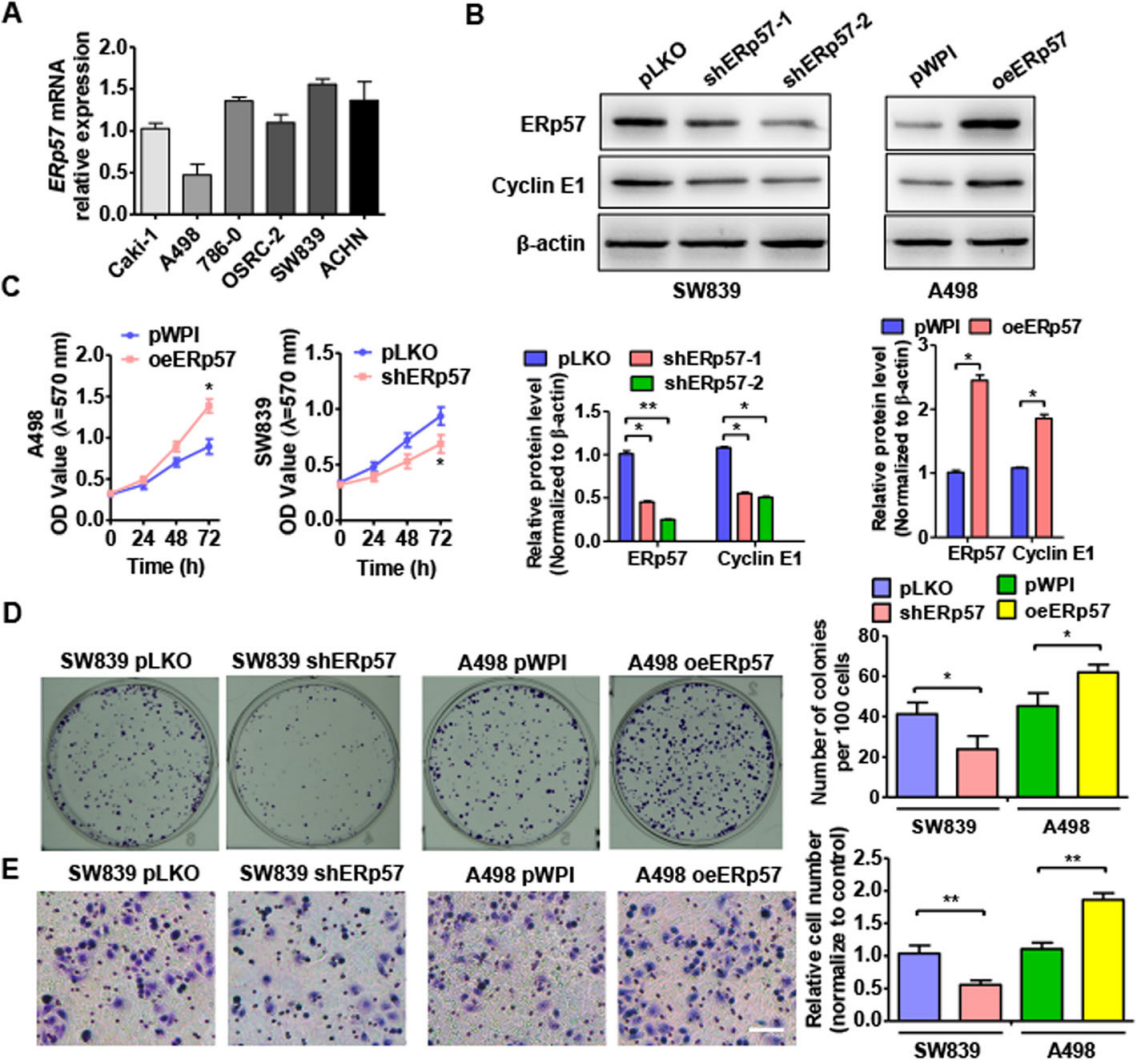
ERp57 plays a critical role in ccRCC cell proliferation and migration. **a** qRT-PCR detected ERp57 mRNA level in 6 ccRCC cell lines (Caki-1, A498, 786–0, OSRC-o, SW839 and ACHN). **b** SW839 cells transfected with shERp57 (shERp57–1 and shERp57–2) or A498 cells transfected with overexpression ERp57 vector (pWPI-ERp57). ERp57 and Cyclin E1 protein level was measured by western blotting. Densitometric analysis from three independent experiments. *P < 0.05, **P < 0.01 vs corresponding control. **c** and **d** Cells were prepared as **b**, cell viability was measured by MTT assay **c** and colony formation assays **d**. Right panel shows the number of colony formation. *P < 0.05, **P < 0.01 vs corresponding control. **e** Cells were treated as (**b**). Transwell migration assay was used to test migration ability. Right panel shows the number of migration cells. **P < 0.01 vs corresponding control

Previous studies have indicated that ERp57 may function as an oncogene to promote the progression of multiple cancers [25, 26]. To investigate the biological functions of ERp57 in ccRCC cell survival, some in vitro loss and gain-of-function experiments were performed. First, we detected relative ERp57 mRNA expression levels in six different ccRCC cell lines (Fig. 2a). qRT-PCR analysis showed that the A498 cell line expressed the lowest levels of ERp57 mRNA compared with the other ccRCC cell lines; therefore, this cell line was selected for gain-of-function experiments. Higher levels of ERp57 mRNA expression were observed in the SW839 cell line; therefore, this cell line was selected for loss-of-function experiments. Next, we knocked down ERp57 in SW839 cells using specific shRNA and overexpressed ERp57 in A498 cells by transfecting cells with a pWPI–ERp57 overexpression vector. Western blotting results showed that transfection of shERp57 led to significantly downregulated levels of ERp57 and the proliferation maker gene, Cyclin E1, in A498 cells compared with the shRNA control vector, while transfection of pWPI–ERp57 in SW839 cells led to upregulated ERp57 and Cyclin E1 protein levels compared with empty vector (Fig. 2b). MTT analysis showed that overexpression of ERp57 promoted A498 cell growth, while suppression of ERp57 inhibited cell proliferation in SW839 cells (Fig. 2c). Colony formation analysis further confirmed these results (Fig. 2d). In addition, transwell migration assay showed that overexpression of ERp57 induced A498 cell migration. Consistently, knockdown of ERp57 in SW839 cells led to markedly reduced cell migration ability (Fig. 2e). Together, these findings suggest that ERp57 functions to promote proliferation and migration in ccRCC cells.

### ILF3 is positively regulated by ERp57

**Fig. 3:**
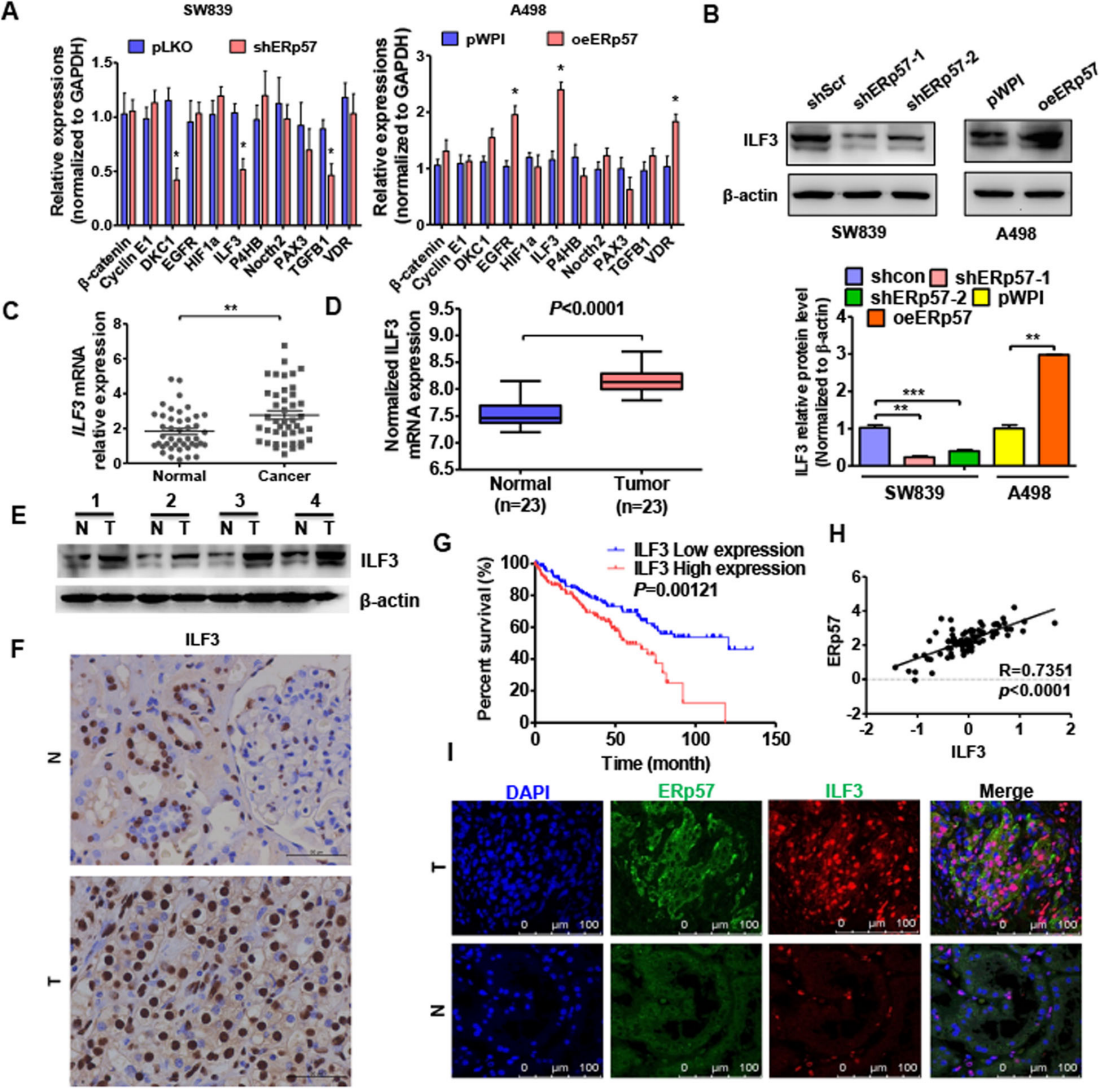
ILF3 is positively regulated by ERp57. **a** SW839 cells transfected with shERp57 or A498 cells transfected with pWP-ERp57. Gene mRNA level was tested by qRT-PCR. *P < 0.05 vs. corresponding control. **b** Cells were prepared as A, IL3 protein level was measured by western blotting. **c** ILF3 mRNA level in ccRCC tissues (*n* = 35) and normal kidney tissues (*n* = 35) was detected by qRT-PCR. **d** ILF3 level from ccRCC tissues and normal kidney tissues was analyzed in TCGA database. **e** and **f** Western blot analysis and immunohistochemistry stain were performed to measure the ILF3 protein level in ccRCC tissues and normal kidney tissues. **g** Kaplan–Meier analysis was used to analyze the overall survival of ccRCC patients with low or high ILF3 level from TCGA database. **h** Pearson correlation analysis was used to analyze the relationship between ILF3 and ERp57 (R = 0.7351, *P* < 0.0001). **i** Immunofluorescence staining was used to locate and detect the expression of ERp57 and ILF3 in A498 cells. Scale bars = 100 μm

To further demonstrate how ERp57 regulates proliferation of ccRCC cells, we selected genes previously reported to be regulated by ERp57 and partially related to cell proliferation. We measured mRNA levels of 11 candidate genes by real-time PCR in the SW839 and A498 cell lines. As shown in Fig. 3a, ILF3 was the only gene that was significantly downregulated in ERp57-depleted SW839 cells, while ILF3 was upregulated in ERp57-overexpressing A498 cells. Western blot analysis showed that overexpression of ERp57 increased ILF3 protein levels; whereas knockdown of ERp57 decreased ILF3 protein levels in ccRCC cells (Fig. 3b). ILF3 expression levels were then measured in ccRCC tissues. The qRT-PCR results showed that ILF3 mRNA levels were significantly higher in ccRCC tissue compared with normal kidney tissues (Fig. 3c). These results were consistent with those found in the TCGA database (Fig. 3d). Additionally, western blot analysis and immunohistochemistry analysis showed that ccRCC tissues had higher protein levels of ILF3 compared with normal kidney tissues (Fig. 3e and f). Additionally, we analyzed the clinical significance of ILF3 in ccRCC, and found that ILF3 mRNA level significantly associated tumor size (Table.1). Survival analysis from the TCGA database showed that higher expression of ILF3 was associated with poor prognosis (Fig. 3g). Therefore, we analyzed the correlation between ERp57 and ILF3 mRNA in ccRCC tissue. We found that ERp57 was positively correlated with ILF3 in ccRCC tissue (Fig. 3h). Immunofluorescence colocalization results showed that high expression levels of ERp57 were associated with high expression of ILF3 (Fig. 3i). Collectively, these results indicate that ERp57 is positively correlated with ILF3, which is also upregulated in ccRCC.

### STAT3 mediates the ERp57 regulating ILF3

**Fig. 4:**
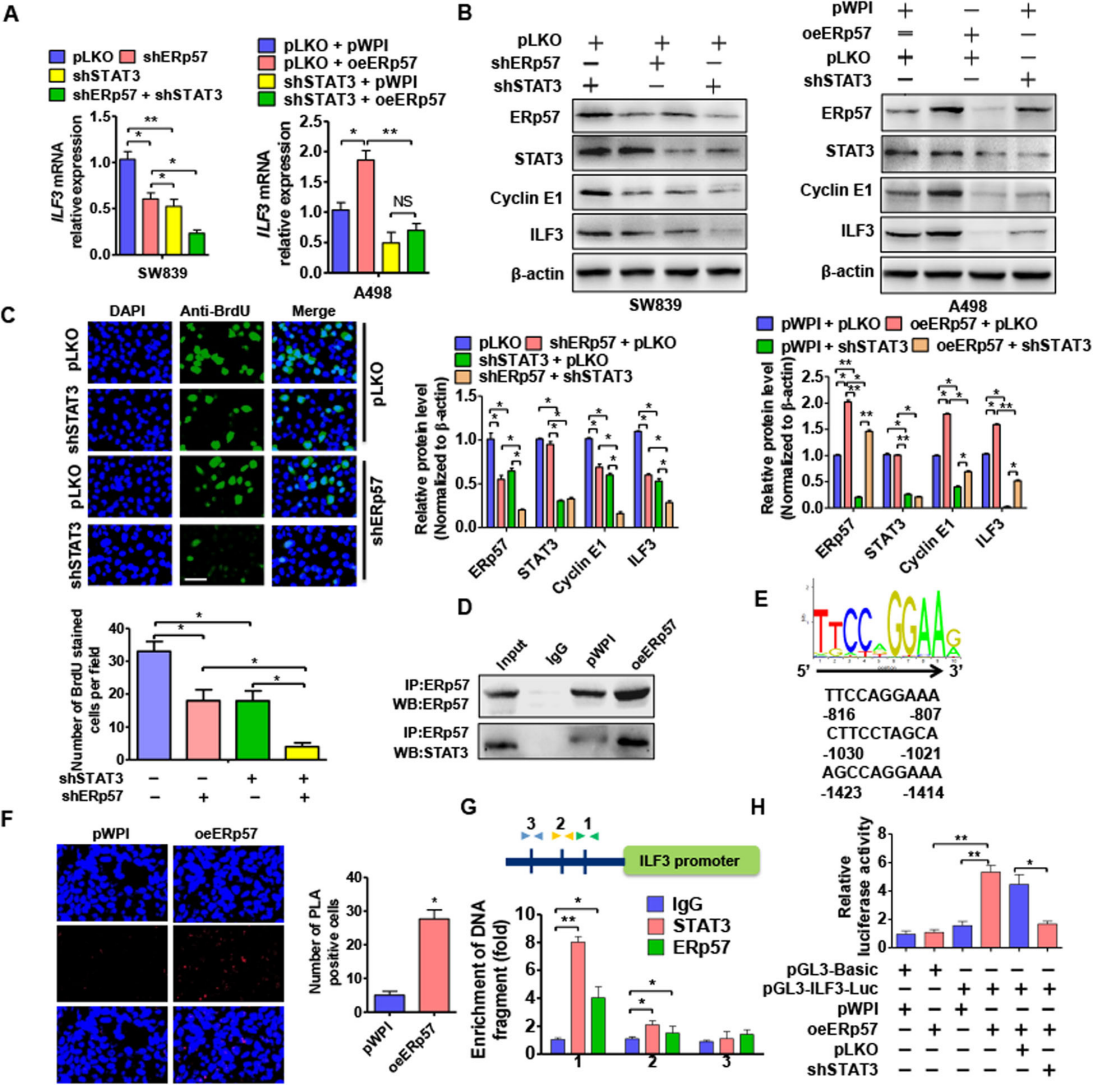
STAT3 mediates the ERp57 regulating ILF3. **a** SW839 cells were transfected shSTAT3 or A498 were cells transfected with STAT3 overexpression vector pWP-STAT3. ILF3 mRNA level was measured by qRT-PCR. *P < 0.05, **P < 0.01 vs. corresponding control. **b** SW839 cells were transfected shSTAT3 or shERp57 respectively or co-transfected them together. A498 cells were transfected with shSTAT3 or pWPI-ERp57 respectively or co-transfected them together. qRT-PCR detected ILF3 mRNA level. *P < 0.05 vs. corresponding control. Western blot performed to detect ERp57, STAT3, ILF3 and Cyclin E1 protein level. **c** SW839 cells were transfected shSTAT3 and shERp57 respectively or transfected them together. Cell proliferation was measured by BrdU stain. Scale bar = 20 μm. Right panel showed analysis for BrdU positive cell number. *P < 0.05 vs corresponding control. **d** A498 cells were transfected with pWPI-ERp57 or empty vector and then cell lysates were immunoprecipitated with antibody against ERp57. Western blot detected ERp57 and STAT3 protein level in lysates. **e** A498 were treated as (**d**), PLA analysis was used to test the interaction between ERp57 and STAT3. Analysis of number of PLA positive cells. *P < 0.05 vs. empty vector. **f** Potential binding site of STAT3 in ILF3 promotor. **g** ChIP-qPCR was used to test STAT3 and ERp57 binding to the ILF3 promoter region in 293A cells. *P < 0.05, **P < 0.01 vs. IgG. **h** ILF3 promoter-luciferase reporter were co-transfected with pWPI-ERp57 or co-transfected pWPI-ERp57 combination of shSTAT3 vector into 293A cells, and then luciferase reporter assays were performed. *P < 0.05 vs. corresponding control

A previous study reported that ERp57 functions as a chaperone protein involved in transcription factor activity. Several studies have indicated that ERp57 binds to STAT3 to form an ERp57/STAT3 complex. This complex is required for the transcriptional activity of STAT3 and some genes downstream of STAT3 [27, 28]. Therefore, we investigated whether STAT3 acted to mediate ERp57 and ILF3. To test this, we first investigated whether STAT3 regulated ILF3 expression in ccRCC cells. We found that suppression of STAT3 expression decreased ILF3 mRNA levels, while enhanced STAT3 expression increased ILF3 mRNA levels in ccRCC cells (Fig. 4a). Rescue experiments further demonstrated that STAT3 mediates the relationship between ERp57 and ILF3. Co-transfection of SW839 cells with shSTAT3 and shERp57 significantly enhanced the inhibitory effects on ILF3 and Cyclin E1 compared with knockdown of STAT3 alone. In contrast, transfection of A498 cells with shSTAT3 reduced ILF3 and Cyclin E1 levels, while this reduction effect could be reversed by co-transfection with ERp57 overexpression vector. Furthermore, as Fig. 4c showed, depletion of STAT3 or ERp57 alone suppressed cell proliferation by using BrdU immunohistochemistry. And these suppression effects could strengthen by knocking down STAT3 and ERp57 together. We next investigated whether ERp57 functioned as a protein partner of STAT3. As expected, co-immunoprecipitation (CoIP) and PLA results confirmed protein–protein interactions between STAT3 and ERp57 (Fig. 4d and f). To further demonstrate the involvement of ERp57 binding with STAT3 in ILF3 transcription, we predicted the putative binding sites of STAT3 in ILF3 promotor fragment. ChIP qPCR analysis and luciferase reporter assays were used to show that STAT3 regulates ILF3 transcription. As shown in Fig. 4g, the ILF3 promoter contains three different sequences that could act as binding sites for STAT3. ChIP PCR results showed both STAT3 and ERp57 proteins could bind to sites 1 and 2, but not site 3. Meanwhile, we performed luciferase reporter assays to determine whether ERp57 combined with STAT3 could promote ILF3 transcription. We found that overexpression of ERp57 markedly increased the activity of the luciferase vector present in the ILF3 promoter. However, luciferase activity remained unchanged after knockdown of endogenous STAT3 combined with transfection of ERp57 overexpression vector (Fig. 4h), suggesting that STAT3 directly cross-linked with the ILF3 promotor. These results indicate that ERp57 interacts with STAT3 and promotes the transcription factor activity of STAT3.

### ILF3 regulates ERp57 by promoting ERp57 mRNA stability

**Fig. 5:**
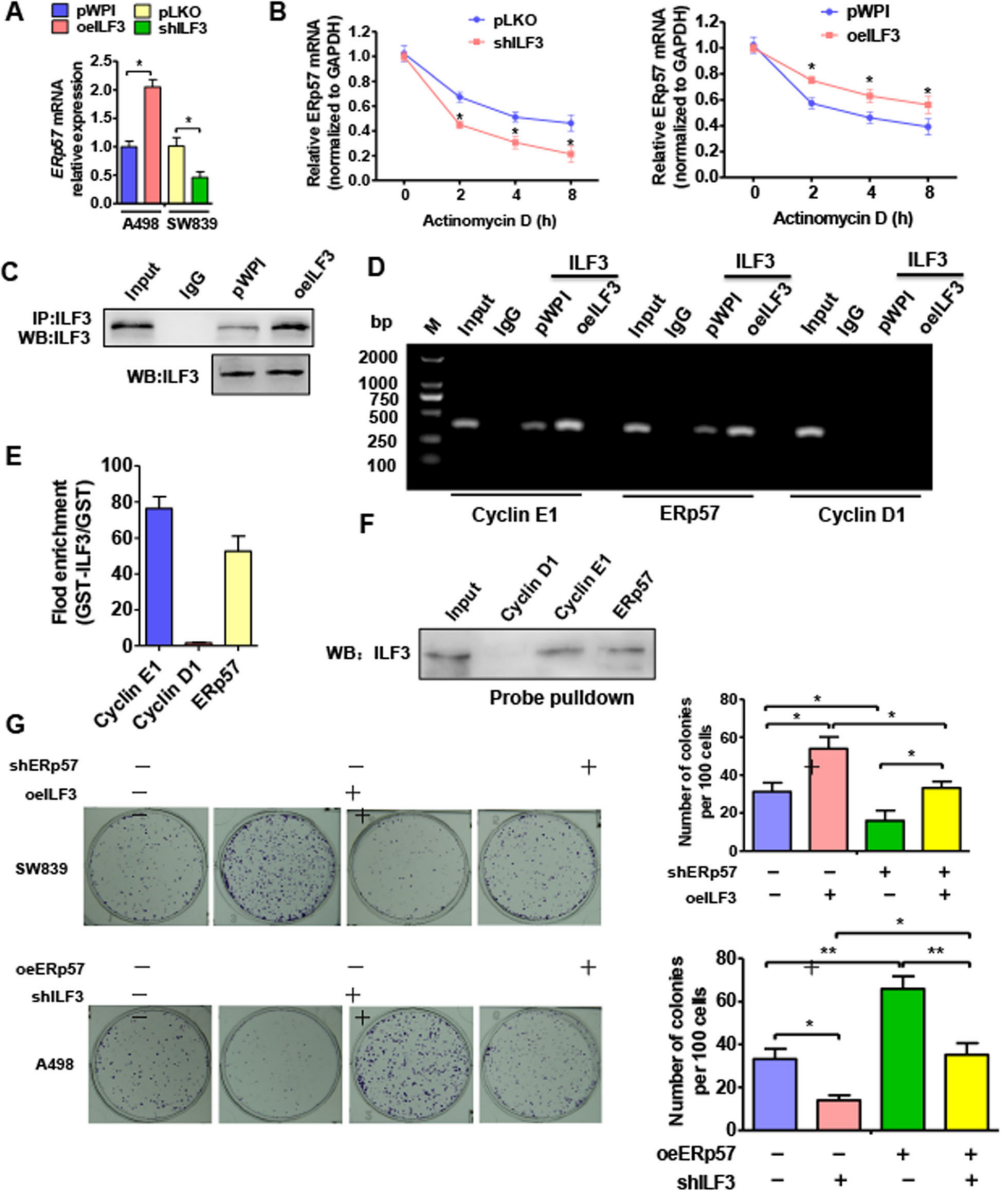
ILF3 regulates ERp57 by promoting ERp57 mRNA stability. **a** SW839 cells transfected with shILF3 or A498 cells transfected with ILF3 overexpression vector pWP-ILF3. ERp57 mRNA level was measured by qRT-PCR. *P < 0.05 vs. corresponding control. **b** Cells were prepared as (**a**) and then exposed to Actinomycin D for 0, 2, 4, and 8 h. ERp57 mRNA level was detected using qRT-PCR. *P < 0.05, vs. corresponding control. **c** A498 cells were transfected with pWPI-ILF3 or empty vector. Western blot was used to measure the precipitation efficiency of ILF3 antibody. (**d** and **e)** RIP PCR and agarose gel electrophoresis was used to test the interaction between ILF3 protein and ERp57 mRNA. **f** A498 cells were transfected with ILF3-probe. Western blot analysis was used to test the ERp57, Cyclin D1, Cyclin E1 in pulldown precipitate. **g** SW839 cells were transfected with shERp57 or pWPI-ILF3 alone, or co-transfected with shERp57 and pWPI-ILF3. A498 cells were transfected with shILF3 or pWPI-ERp57 alone or co-transfected them together. Colony formation assay tested cell ability of proliferation. Right panel shows the analysis of colony formation number. *P < 0.05, ** *P <* 0.01 vs. corresponding control

Since ERp57 and ILF3 were positively correlated in ccRCC and ERp57 moderated ILF3 expression, we investigated whether ILF3 regulates ERp57 expression in ccRCC. Surprisingly, overexpression of ILF3 promoted ERp57 mRNA levels, while knockdown of ILF3 inhibited ERp57 mRNA level in ccRCC cells (Fig. 5a). As an RNA-binding protein, ILF3 has been reported to bind to the Cyclin E1 3′UTR, leading to mRNA stabilization. Therefore, we explored whether ILF3 could bind to ERp57 mRNA and moderate its stability. We used qRT-PCR analysis to measure changes in ERp57 mRNA levels over time after blocking transcription with Actinomycin D (ActD). We found that depletion of ILF3 in SW839 cells inhibited the effects of ActD on ERp57 mRNA stability, whereas ILF3 overexpression promoted the effects of ActD, indicating that ILF3 promoted ERp57 expression by enhancing its mRNA stability (Fig. 5b). To further explore this, we investigated whether ILF3 could bind to the ERp57 mRNA 3′UTR. We performed an in vitro RNA pull-down assay and a RNA immunoprecipitation assay (RIP). The CoIP results showed that the ILF3 antibody could pull down sufficient levels of endogenous ILF3 protein (Fig. 5c). RIP-PCR analysis showed that Cyclin E1 and ERp57 mRNA 3′UTR, but not Cyclin D1, were present in the protein–RNA complex pulled down by the ILF3 antibody (Fig. 5d and e). Consistently, biotin oligo pull-down also revealed that precipitates from the ERp57 mRNA-3′UTR and positive control Cyclin E1-mRNA probes contained ILF3 protein, but the Cyclin D1 mRNA-3′UTR probe did not pull down ILF3 protein (Fig. 5d and f). These results suggested that ILF3 could bind to ERp57 mRNA 3′UTR and enhance its mRNA stability. We then used colony forming assays to determine whether ILF3 was involved in ERp57-induced ccRCC cell proliferation. The results showed that ccRCC cell proliferation was increased by ILF3 overexpression, but decreased by depletion of ILF3 (Fig. 5g, line 2). Moreover, overexpression of ILF3 combined with knockdown of ERp57 reversed the increase in cell proliferation induced by ILF3. However, the decrease in cell proliferation due to depletion of ILF3 could only be weakened by overexpression of ERp57. These findings strongly support that ILF3 is involved in ERp57 regulation of ccRCC cell proliferation.

### ERp57/STAT3 /ILF3 feedback loop plays a key role in ccRCC cell proliferation

**Fig. 6:**
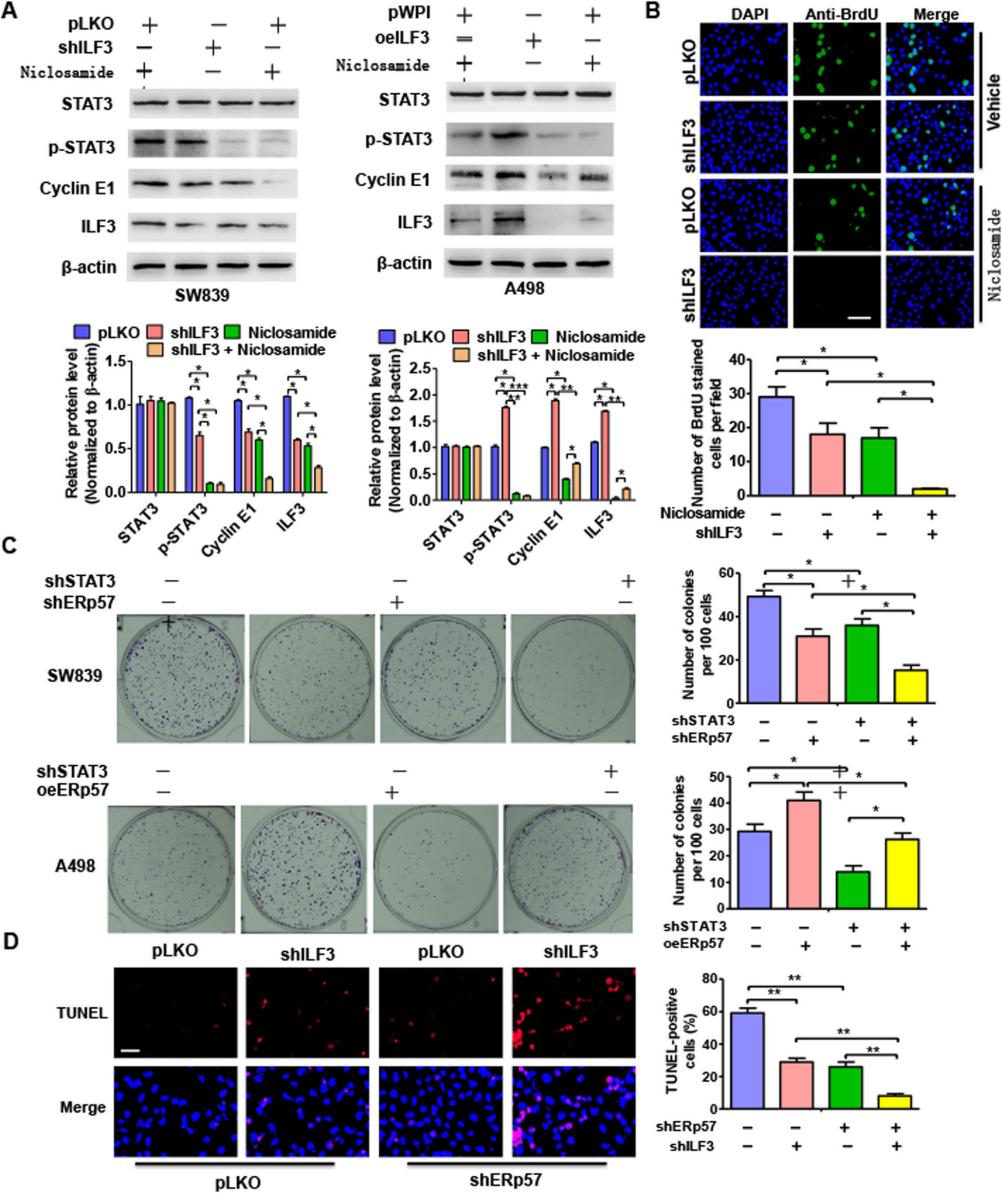
ERp57/STAT3/ILF3 feedback loop plays a key role in ccRCC cell proliferation. **a** SW839 cells were transfected with shILF3 or sh-control then cells were treated with niclosamide or not. A498 cells were transfected with pWPI-ILF3 or empty vector then cells were treated with niclosamide or not. STAT3, p-STAT3, Cyclin E1 and ILF3 protein levels were measured by western blotting. **b** SW839 cells were treated as (**a**), BrdU stain was used to test cell viability. Scale bar = 20 μm. Bottom panel showed analysis for BrdU positive cell number. *P < 0.05, vs corresponding control. **c** SW839 cells were transfected with shILF3 or shSTAT3 alone, or co-transfected with them together. A498 cells were transfected with pWPI-ILF3 or shSTAT3 alone, or co-transfected with them together. Colony formation assay measured cell ability of proliferation. Right panel shows the analysis of colony formation number. *P < 0.05, vs. corresponding control. **d** SW839 cells were transfected with shILF3 or shERp57 alone, or co-transfected with them together. Cell apoptosis was tested by TUNEL Tunnel apoptosis analysis. Right panel showed analysis for BrdU positive cell number. ** *P* < 0.01 vs. corresponding control

To elucidate whether the ERp57/STAT3/ILF3 feedback loop is involved in ccRCC cell proliferation, we preformed rescue experiments. As shown in Fig. 6a, SW839 and A498 cells treated with the STAT3 inhibitor, niclosamide, showed decreased levels of phosphorylated STAT3 (p-STAT3), Cyclin E1, and ILF3, but showed no effect on total STAT3 protein levels. This inhibitory effect could be enhanced by simultaneous knockdown of ILF3 in SW839 cells and reversed by overexpression of ILF3 in A498 cells. Moreover, BrdU staining showed that niclosamide treatment of SW839 cells reduced BrdU-positive cells, and this effect could be enhanced by silencing ILF3 (Fig. 6b). Similarly, transfection of ccRCC cells with shSTAT3 inhibited cell growth compared with negative control, as determined by colony formation assays. Co-transfection of A498 cells with shSTAT3 and shILF3 enhanced the inhibitory effects, whereas overexpression of ILF3 increased SW839 cell growth induced by depletion of STAT3 (Fig. 6c). In addition, as shown in Fig. 6d, knockdown of ILF3 or ERp57 significantly inhibited cell apoptosis tested by TUNEL assay. Further, these inhibition effects could be enhanced by depleted them together. Together, these data demonstrate that the ERp57/STAT3/ILF3 axis plays an essential role in ccRCC cell proliferation.

### Intervention of ILF3/ERp57/STAT3 axis inhibits ccRCC xenograft growth in vivo

**Fig. 7:**
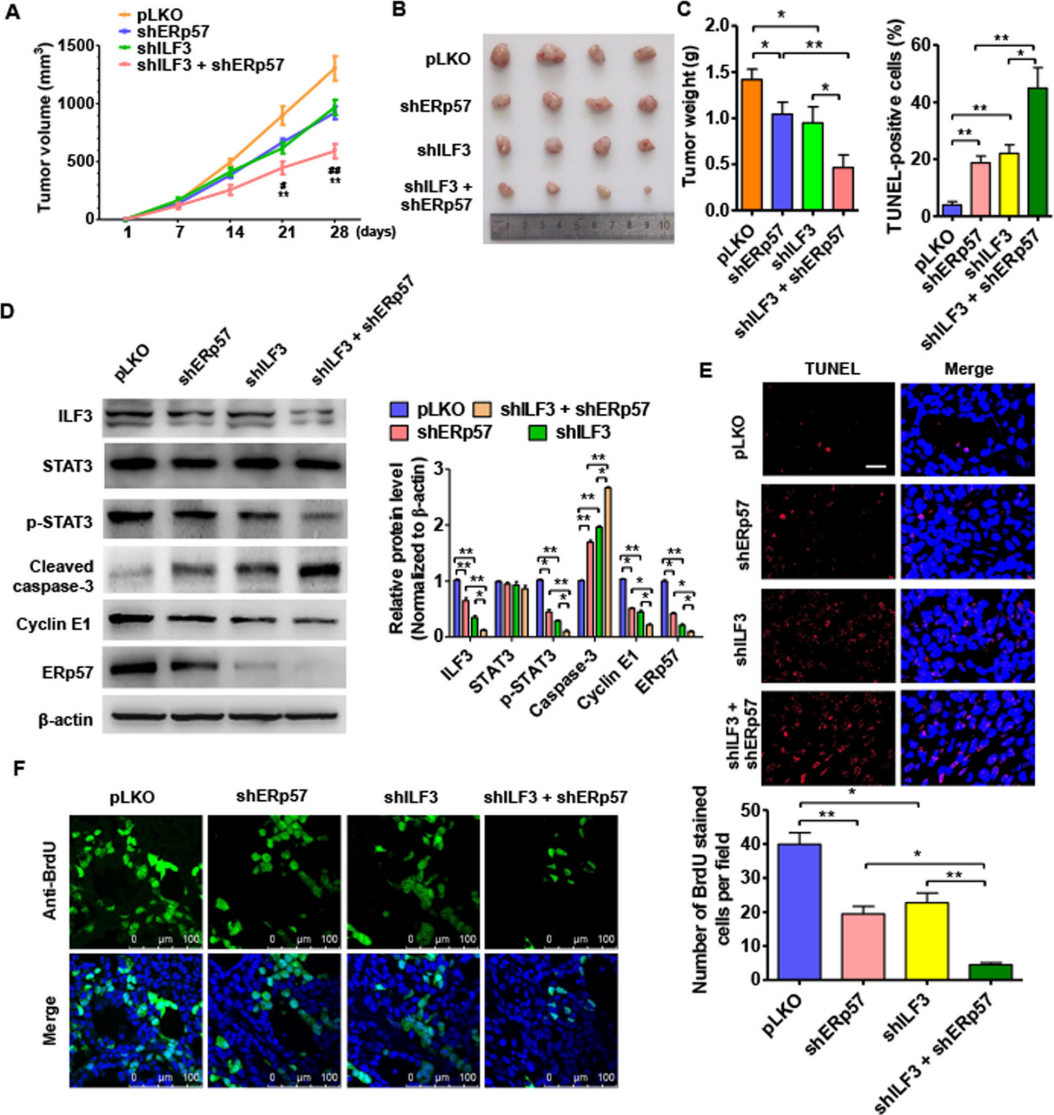
Intervention of ILF3/ERp57/stat3 axis inhibits ccRCC xenograft growth in vivo*.* **a** SW839 cells engineered to stably knockdown of ERp57, knockdown of ILF3 or knockdown of ERp57 and ILF3 together then the cells were injected subcutaneously to the nude mice to establish ccRCC xenograft tumors. Tumor volumes were monitored by direct measurement. **b** Representative tumor sizes in each group of mice. **c** Xenograft tumor wet weight in each group of mice. *P < 0.05, **P < 0.01 vs. corresponding control. **d** Western blot analysis was used to measure STAT3, p-STAT3, ERp57, ILF3, Cyclin E1 and Cleaved caspase-3 protein level in xenograft tumor. **e** Tunnel apoptosis analysis was used to test apoptosis in xenograft tumor. Left panel shows the tunnel-positive cell number. *P < 0.05, **P < 0.01 vs. corresponding control. **f** BrdU stain was used to test cell viability. Scale bar = 20 μm. Right panel showed analysis for BrdU positive cell number. *P < 0.05, ** *P* < 0.01 vs. corresponding control

**Fig. 8:**
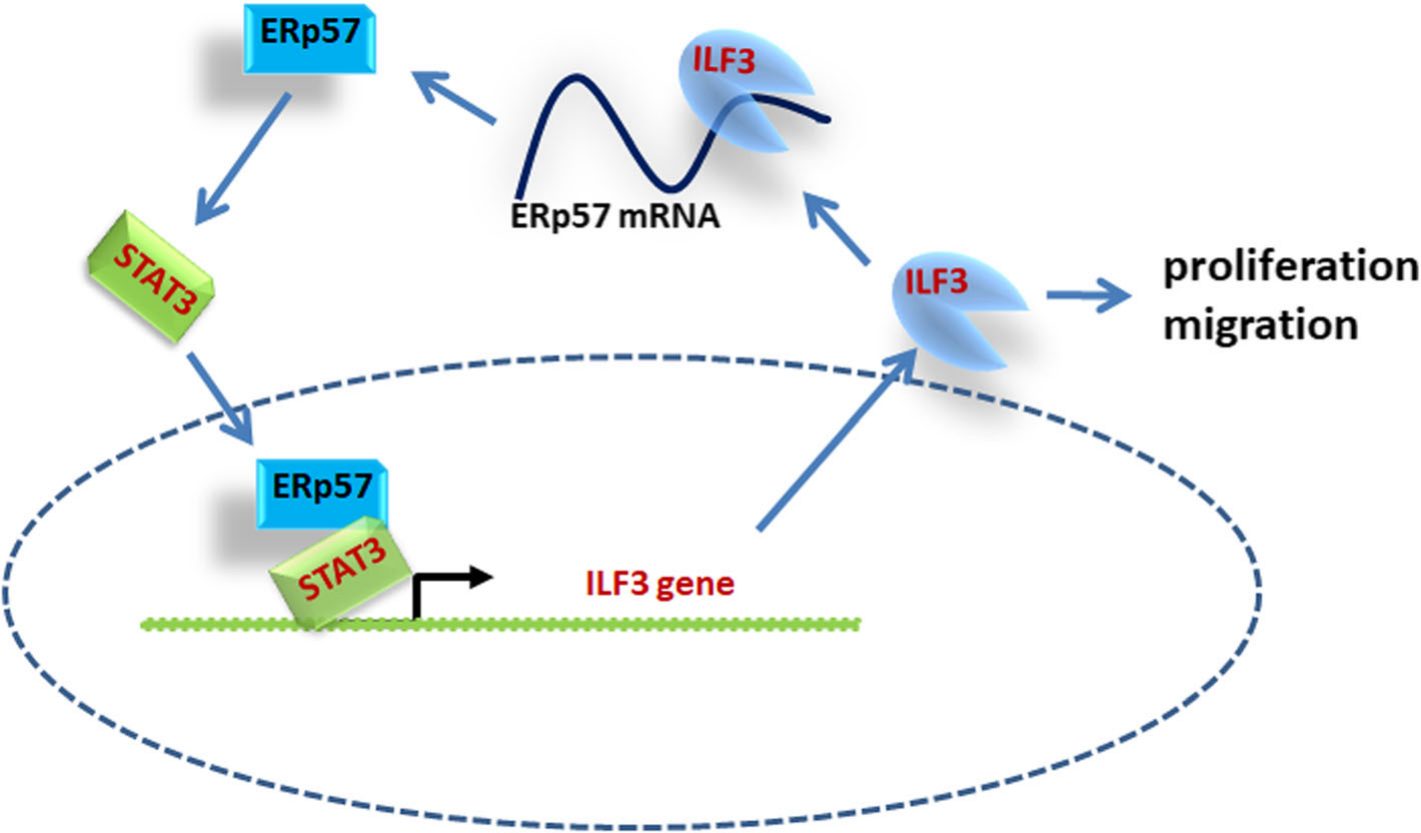
Proposed model for ERp57/STAT3/ILF3 in ccRCC feedback loop in regulating ccRCC progression

We used a ccRCC nude mouse xenograft model to corroborate our findings that ERp57 and ILF3 were involved in ccRCC cell growth in an in vivo model. To test this, SW839 cells with stably depleted ERp57 or ILF3 alone, or knockdown of both were implanted into nude mice. As expected, tumor volumes were significantly decreased in the shERp57 and shILF3 groups compared with vehicle control group. Furthermore, the tumor volume was much smaller in mice implanted with cells with simultaneous knockdown of ERp57 and ILF3 compared with those with depletion of either ERp57 or ILF3 alone (Figs. 7a and b). Consistent with these findings, the mean wet weights of the tumors were significantly lower in mice with combined knockdown of ERp57 and ILF3 compared with those with knockdown of either ERp57 or ILF3 alone (Fig. 7c). Western blot analysis demonstrated that silencing of either ERp57 or ILF3 alone significantly downregulated levels of p-STAT3, ERp57, ILF3, and Cyclin E1, and was accompanied by an increase in cleaved caspase-3 expression compared with vehicle control. These effects could be enhanced by combined knockdown of ERp57 and ILF3 (Fig. 7d). TUNEL staining was used to measure cell apoptosis in xenograft tumors and showed that depletion of either ERp57 or ILF3 promoted apoptosis and was enhanced by combined suppression of ERp57 and ILF3 (Fig. 7e). Furthermore, depletion of ERp57 or ILF3 could decreased the number BrdU positive cell while these effects cloud be enhanced by knocking down them together, indicating that downregulated ERp57 or ILF3 inhibited cell proliferation in vivo (Fig. 7f). As shown in Additional file 1: Figure S1 A, SW839 cells with stably depleted shSTAT3 were implanted into nude mice. The tumor volumes were significantly decreased in the shSTAT3 groups compared with vehicle control group, indicating that knockdown of shSTAT3 inhibited ccRCC cell growth in vivo. As shown in Additional file 1: Figure S1, SW839 cells with stably depleted shSTAT3 were implanted into nude mice. The tumor volumes were significantly decreased in the shSTAT3 groups compared with vehicle control group, indicating that knockdown of shSTAT3 inhibited ccRCC cell growth in vivo*.* These data also suggested that ILF3 and ERp57 regulate ccRCC proliferation. Figure 8 shows our proposed model illustrating the role of the ERp57/STAT3/ILF3 feedback loop in ccRCC.

## Discussion

In present study, we reported an investigation of biological role of ERp57/STAT3/ILF3 feedback loop in the moderation of ccRCC oncogenesis. First, ERp57 expression was found to be significantly increased in ccRCC tissues in the TCGA database and in clinical samples, and higher levels of ERp57 in patients were correlated with poor prognosis. Second, ERp57 was shown to function as an oncogene, promoting ccRCC proliferation and migration in vivo and in vitro. Third, ERp57 positively regulated ILF3 expression. Mechanically, ERp57 binds to STAT3, which acts as an ILF3 transcription factor to form a stable complex that can activate downstream transcription factors. Additionally, ILF3 may could enhance ERp57 mRNA stability by binding to ERp57 mRNA, suggesting that ERp57/STAT3/ILF3 plays a growth promotion in ccRCC.

ERp57 is a key member of the PDI family. Similar to disulfide isomerase, ERp57 is localized in the ER and plays a direct role in protein folding and an indirect role in major histocompatibility complex heavy chain loading [29, 30]. However, unlike traditional PDI family members that are only localized in ER, ERp57 was also found in various other subcellular locations, such as the, cytosol, mitochondria, and the membrane [31–34]. Due to the characteristics of its localization, ERp57 plays a critical role in binding proteins to mediate the signaling pathway activation [35]. There is growing evidence to show dysregulation of ERp57 in various malignant cells including ovarian cancer [12], breast cancer [36], melanoma [34], laryngeal cancer [27] and leukemia [37]. For example, depletion of ERp57 in breast cancer cell reduces cell proliferation by regulating PERK-mediated activation of the unfolded protein response [25]. Moreover, ERp57 affects mTORC1 activation by interacting with mTOR, and is associated with cell proliferation [38]. In the present study, we first confirmed significantly higher levels of ERp57 in ccRCC tissue compared with normal kidney tissue. We further analyzed the relationship between ERp57 levels and patient outcome and found that higher levels of ERp57 in ccRCC patients were associated with poor prognosis. Our screening identified that knockdown of ERp57 inhibited cell proliferation of ccRCC in vivo and in vitro, indicating that ERp57 plays a role in the promotion of ccRCC cell proliferation.

Signal transducer and activator of transcription 3 (STAT3) is a member of STAT family that regulates the transcription of responsive genes involved in a variety of critical functions, including cell differentiation, proliferation, apoptosis and angiogenesis [39, 40]. There is abundant evidence to show that elevated levels or abnormal activation of STAT3 contribute to cancer cell proliferation, including breast cancer [41], prostate cancer [42], colon cancer [43], and leukemia [44]. Qin showed that p-STAT3 was overexpressed in patients with ccRCC, and high expression of nuclear p-STAT3 was associated with poor patient survival. Moreover, increased expression of pSTAT3 was also shown to be correlated with poor tumor features, such as high tumor grade, large tumor size, and advanced T stage and AJCC stage [45]. However, STAT3 does not act alone, and co-activators such as CBP/p300, c-jun, Oct-1, and NcoA/SRC1a have been confirmed to be associated with this transcriptional factor [46–48]. Studies have demonstrated that ERp57 could bind to STAT3 in the cytoplasm and facilitate STAT3 shuttling cytosol to the nucleus [49, 50]. In addition, nuclear ERp57 functions as a chaperone that binds to DNA and enhances DNA-binding of the STAT3 complex to activate proximal transcription factors [51]. In the present study, we revealed that ERp57 could interact with STAT3 and form a complex. The complex simultaneously cross-linked to the ILF3 DNA promoter and enhanced ILF3 transcription. Importantly, we confirmed that ERp57 is essential for STAT3 transcriptional activation of ILF3. Further studies are required to determine whether ERp57 plays a role in STAT3 transportation from the cytoplasm to the nucleus.

STAT3 regulates various target genes that have different effects on tumorigenesis. Several recent studies have shown that STAT3 transcriptionally regulates key tumor-driving genes that are essential for tumor pathogenesis, such as TNFRSF1A [52], MCY and SOX1 [53]. In addition, STAT3 may interact with the non-coding RAN promoter to facilitate its transcription [54]. In the present study, we revealed that STAT3 directly promoted ILF3 gene as a transcription factor, and that ILF3 levels were dependent on STAT3 activation. Both shRNA-mediated depletion of STAT3 and reduction of p-STAT3 via a molecular inhibitor decreased ILF3 levels in ccRCC cells. Furthermore, STAT3 regulation of ILF3 was involved in ccRCC proliferation. Importantly, we found that niclosamide could reduce ccRCC cell growth in vitro by targeting the STAT3/ILF3 axis, indicating its antitumor effect in ccRCC. However, further in vivo experiments and clinical trials are required to confirm its effects in the clinical treatment of ccRCC.

ILF3, also as known as NF90, NF110, and CBTF in humans, is an important double-stranded RNA-binding protein generated via splicing of the *ILF3* gene [55]. By binding to various RNAs, ILF3 is considered to be involved in almost all steps of RNA metabolism, from transcription to degradation. For example, as a transcription factor co-activator, ILF3 together with NF45 binds to a CTGTT sequence and promotes human breast tumor progression by regulating uPA expression [19]. ILF3 interacts with the transcription factor, p54, to assist p54-mediated promotion of the human survivin gene expression that plays a key role in tumor proliferation and apoptosis [56]. As a translational regulator, ILF3 is involved in retaining cellular transcripts in the nucleus and controlling their export into the cytoplasm and promotes protein translation [57]. As a promoter of mRNA stabilization, ILF3 may bind to the AG-rich element of a gene’s 3′UTR to enhance mRNA stability [58]. On the other hand, ILF3 blocks the binding site of degradation factors to prevent mRNA degradation, leading to mRNA stabilization [59]. In the present study, we first confirmed that ILF3 positively regulated ERp57 expression. We first demonstrated that ILF3 acted as an RNA-binding protein and could bind to the ERp57 mRNA 3′UTR to stabilize ERp57 mRNA. This demonstrated that ILF3/ERp57 formed a positive feedback loop mediating STAT3, and ILF3/STAT3/ERp57 together promoted ccRCC proliferation. Analysis of clinical samples and the TCGA database showed that higher ILF3 levels in patients was correlated with poor survival, indicating that ILF3 may function as a clinical biomarker of prognosis. However, studies using larger numbers of clinical samples are required to further evaluate its clinical value.

Importantly, we revealed a feedback loop regulation which involved in ccRCC proliferation in the present study. In general, ERp57 as a protein partner, interacts with STAT3, forming a complex which transcriptionally regulates ILF3 expression. In contrast, ILF3 binds to ERp57 3’UTR and positively regulate ERp57 expression by enhancing its mRNA stability. The mutual promotion effect of ERp57 and ILF3 participates in ccRCC progression.

## Conclusion

In summary, the present study demonstrated that dysregulation of ERp57 enhanced ccRCC cell survival by initiating a STAT3/ILF3 feedback loop that was correlated with prognosis in patients with ccRCC. These findings provide useful insight into the regulation of tumor cell metabolism by ERp57/STAT3/ILF3 in ccRCC as a potential therapeutic target.

## Supplementary information

**Additional file 1: Figure S1.** SW839 cells engineered to stably knockdown of STAT3 then the cells were injected subcutaneously to the nude mice to establish ccRCC xenograft tumors. Tumor volumes were monitored by direct measurement.

## Data Availability

Not applicable.

## Abbreviations

ccRCC: Clear cell renal cell carcinoma
ChIP: Chromatin immunoprecipitation
CoIP: Co-immunoprecipitation
IFN3: Interleukin enhancer-binding factor 3
PDI: Protein disulfide isomerase
PLA: proximity ligation assay
RIP: RNA immunoprecipitation
STAT3: Signal transducer and activator of transcription 3
TCGA: The Cancer Genome Atlas;
uPA: urokinase-type plasminogen activator
UTR: untranslated region
