# Supplementary material for: Upregulation of ERp57 promotes clear cell renal cell carcinoma progression by initiating a STAT3/ILF3 feedback loop
Source: J Exp Clin Cancer Res. 2019 Oct 30;38:439. doi: 10.1186/s13046-019-1453-z (PMC6864981; doi:10.1186/s13046-019-1453-z)
Supplement: Supplementary file 1 — Additional file 1: Figure S1. SW839 cells engineered to stably knockdown of STAT3 then the cells were injected subcutaneously to the nude mice to establish ccRCC xenograft tumors. Tumor volumes were monitored by direct measurement. [file 13046_2019_1453_MOESM1_ESM.docx]

**Additional file**

**pLKO**

**shSTAT3**


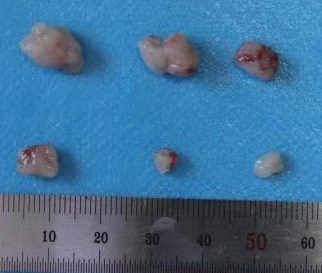


**Figure S1.** SW839 cells engineered to stably knockdown of STAT3 then the cells were injected subcutaneously to the nude mice to establish ccRCC xenograft tumors. Tumor volumes were monitored by direct measurement.
